# Procedural and Antithrombotic Therapy Optimization in Patients with Atrial Fibrillation Undergoing Percutaneous Coronary Intervention: A Narrative Review

**DOI:** 10.3390/jcdd12040142

**Published:** 2025-04-08

**Authors:** Domenico Simone Castiello, Federica Buongiorno, Lina Manzi, Viviana Narciso, Imma Forzano, Domenico Florimonte, Luca Sperandeo, Mario Enrico Canonico, Marisa Avvedimento, Roberta Paolillo, Alessandra Spinelli, Stefano Cristiano, Fiorenzo Simonetti, Federica Semplice, Dario D’Alconzo, Donato Maria Vallone, Giuseppe Giugliano, Alessandro Sciahbasi, Plinio Cirillo, Felice Gragnano, Paolo Calabrò, Giovanni Esposito, Giuseppe Gargiulo

**Affiliations:** 1 Department of Advanced Biomedical Sciences, University of Naples Federico II, 80138 Naples, Italy; ds.castiello@gmail.com (D.S.C.); federicabuongiorno2@gmail.com (F.B.); lina.manzi93@gmail.com (L.M.); viviana.narciso@gmail.com (V.N.); imma.forzano@gmail.com (I.F.); florimontedomenico@gmail.com (D.F.); luca.sperandeo95@gmail.com (L.S.); marioenrico.canonico@unina.it (M.E.C.); m.avvedimento@gmail.com (M.A.); robe.paolillo@gmail.com (R.P.); alessandra.spinelli@unina.it (A.S.); stefano.cristiano@unina.it (S.C.); fiorenzosimonetti@gmail.com (F.S.); federicasemplic@gmail.com (F.S.); dario.dalconzo@gmail.com (D.D.); donatovallone@outlook.it (D.M.V.); giuseppe.giugliano@unina.it (G.G.); pcirillo@unina.it (P.C.); espogiov@unina.it (G.E.); 2 Interventional Cardiology, Sandro Pertini Hospital, 00157 Rome, Italy; asciahbasi@gmail.com; 3 Department of Translational Medical Sciences, University of Campania “Luigi Vanvitelli”, 80138 Naples, Italy; felice.gragnano@unicampania.it (F.G.); paolo.calabro@unicampania.it (P.C.); 4 Division of Cardiology, Azienda Ospedaliera di Rilievo Nazionale Sant’Anna e San Sebastiano, 81100 Caserta, Italy

**Keywords:** atrial fibrillation, percutaneous coronary intervention, antiplatelet, oral anticoagulant, intracoronary imaging, vascular access, bleeding, thrombosis

## Abstract

In the past decades, percutaneous coronary intervention (PCI) has become the most common modality for myocardial revascularization in patients with coronary artery disease (CAD). Dual antiplatelet therapy (DAPT) with aspirin and a P2Y12 inhibitor is essential in all patients undergoing PCI to prevent thrombotic complications. A large proportion of patients undergoing PCI also have concomitant atrial fibrillation (AF), thus requiring an oral anticoagulant (OAC) to prevent ischemic stroke or systemic embolism. However, the association between OAC and DAPT further increases the risk of bleeding. Compared with a triple antithrombotic therapy (TAT), dual antithrombotic therapy (DAT) has shown to reduce bleeding events, but at the cost of higher risk of stent thrombosis. In this field, patients with AF undergoing PCI represent a special population with significant challenges, and several strategies are needed to reduce the risk for bleeding complications. In this review, we will discuss both the procedural and antithrombotic strategies to optimize ischemic and bleeding outcomes in patients with AF undergoing PCI.

## 1. Introduction

**Figure 1:**
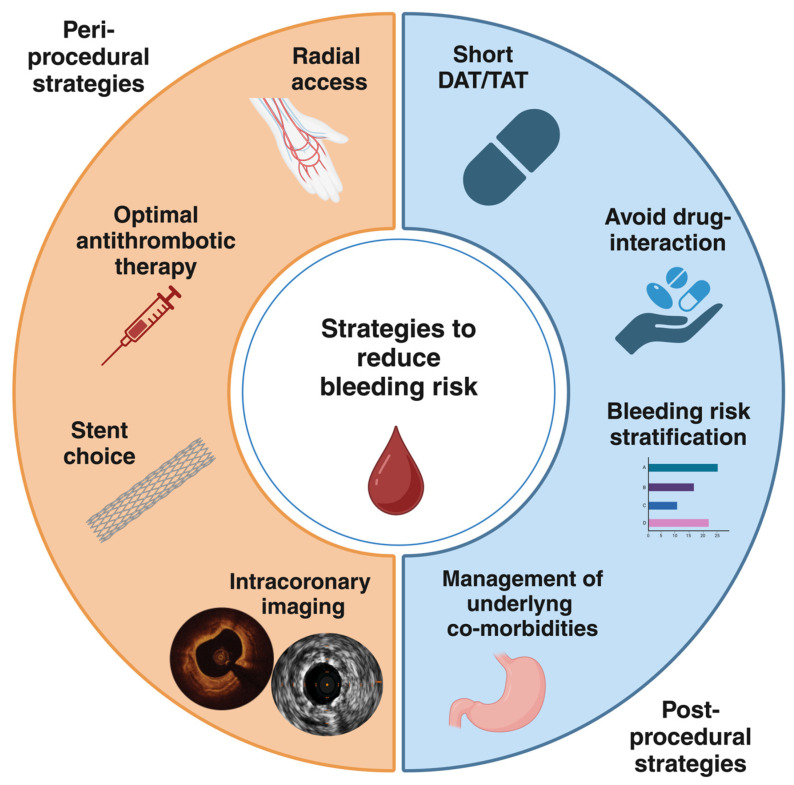
Peri-procedural and post-procedural strategies to reduce bleeding risk in patients requiring OACs undergoing PCI. DAT, dual antithrombotic therapy; TAT, triple antithrombotic therapy.

Coronary artery disease (CAD) still remains the leading cause of morbidity and mortality worldwide [1]. Atrial fibrillation (AF) is a highly prevalent arrhythmia that increases with age [2]. Notably, the prevalence of both CAD and AF increases with advancing age, and these conditions often coexist. Up to 40% of patients with AF also have CAD, many of whom require coronary revascularization, most commonly with percutaneous coronary intervention (PCI). On the other hand, around 10% of patients undergoing PCI also have AF [3]. Dual antiplatelet therapy (DAPT) is the mainstay treatment following PCI with drug-eluting stents (DES) implantation in order to prevent thrombotic complications, specifically stent thrombosis (ST) and target lesion failure (TLF) [4,5]. However, patients with AF require oral anticoagulant (OAC) for the prevention of ischemic stroke or systemic embolism. Therefore, patients with AF undergoing PCI represent a special population with significant challenges for the choice of optimal antithrombotic treatment. Indeed, anticoagulant and antiplatelet therapies are required with a subsequent significant increase in bleeding risk [6,7,8,9,10]. In recent years, bleeding events have been recognized as having prognostic relevance nearly equal to ischemic events, with a significant impact on mortality [11,12,13]. For this reason, a careful evaluation of bleeding risk is mandatory in all patients undergoing PCI and, even more, in the subgroup of those with AF. Several scores have been developed to define patients at high bleeding risk (HBR) and standardize their definition in clinical practice [4,14,15,16]. In this respect, less intensive antithrombotic strategies have been explored to reduce the bleeding risk [17,18,19,20,21]. Procedural aspects are crucial as well to optimize ischemic and bleeding outcomes in this complex cohort of patients. In this review, we will analyze the evidence regarding the procedural choices to achieve optimal stenting results. We will summarize the most recent data about antithrombotic therapies in patients with AF undergoing PCI. Furthermore, we will discuss both peri-procedural and post-procedural potential strategies to reduce bleeding risk (Figure 1). For this purpose, we searched on PubMed all relevant studies, reviews, meta-analyses, international guidelines, expert consensus documents, and their references to provide a comprehensive overview on this topic.

## 2. Peri-Procedural Strategies

In recent years, the implementation of several important developments in PCI has been observed in every step of the procedure. In this respect, improved efficacy and safety were achieved in terms of ischemic and bleeding outcomes for patients undergoing PCI [22]. Hence, PCI has become the most common modality for myocardial revascularization with a significant increase in its use over coronary artery bypass graft (CABG). Several strategies have contributed to optimize outcomes in patients undergoing PCI. These developments are pivotal also in the complex cohort of patients with AF. The optimization strategies obviously start with appropriateness of indication to invasive diagnostic and revascularization procedures in order to avoid exposing patients to both ischemic and bleeding risks related to the procedure and pharmacotherapy. Said that, there are some procedural aspects to be accounted for, particularly in AF patients requiring OACs, to avoid bleeding but also thrombotic complications because patients with bleeding may require antithrombotic drugs de-escalation or interruption [23].

### 2.1. Antithrombotic Therapy Before and During PCI

The choice of antithrombotic therapy is crucial before, during, and after the procedure in order to limit the risk of bleeding, particularly in patients requiring OACs and even more in those who are already under OAC therapy. In the acute setting, the guidelines from the European Society of Cardiology (ESC) have downgraded the recommendation to pretreatment (administration of an oral P2Y12 inhibitor in addition to aspirin) in patients with ST-elevation myocardial infarction (STEMI) and have contraindicated routine pretreatment in non-ST-elevation acute coronary syndrome (NSTE-ACS) patients [24]. However, even in STEMI patients, particularly those on chronic therapy with OACs, it could be reasonable to avoid pretreatment. Indeed, in the modern era of efficient treatment networks, the patients arrive to catheterization laboratory in short times from first medical contact, and it is well established that, in the acute phase, the start of the antiplatelet effect of oral P2Y12 inhibitors is delayed [24,25]. In both the chronic and acute settings, the choice of antiplatelet therapy should not include ticagrelor or prasugrel instead of clopidogrel when an OAC is indicated. Parenteral anticoagulation is also crucial. Weight-adjusted unfractionated heparin (UFH) continues to be the most commonly used and recommended parenteral anticoagulant. Bivalirudin use has been reduced considerably in many countries for different reasons, including high cost, preliminary evidence of higher rates of stent thrombosis than UFH, the greater use of transradial access (TRA) leading to wrongly perceiving a lower need of reducing access-site bleeding, the lack of a reversal agent, and the gradual downgrade in clinical guideline recommendations. However, several recent data have showed that bivalirudin can be associated with reduced bleeding complications compared with UFH, including access site-related and non-access site-related bleedings, without increasing risk of thrombotic complications when used with a prolonged infusion at a full dose [24,26,27,28,29,30,31]. Therefore, it should not be excluded that selected patients, such as those at higher risk of bleeding as those with AF undergoing PCI, might still benefit from using bivalirudin and that future recommendations might reconsider its use in selected patients, such as those at HBR.

### 2.2. Vascular Access

The choice of vascular access is the first step in every PCI procedure and plays a key role in outcomes of patients with HBR undergoing PCI, including patients with AF. Indeed, even if the improvement of PCI techniques and antithrombotic therapies have lowered the risk of ischemic events, a significant increase in bleeding and vascular complications has been observed [32]. Access-site related bleeding, although rarely fatal, is a frequent complication after PCI, which affects the duration of hospitalization, costs, and quality of life [33]. Among patients undergoing PCI, access site-related bleeding accounts for 30–70% of total bleeding events with prognostic implications [34]. Historically, transfemoral access (TFA) was the first and most used vascular access to perform coronary angiography and subsequently PCI, but its use was a common site of bleeding events [35]. In recent years, TRA use increased worldwide to perform invasive procedures, including PCI. Compared with TFA, the TRA is more superficial and has a smaller caliber. Consequently, TRA is technically more demanding, but makes access site hemostasis more predictable with the opportunity to reduce access-related bleeding events [36].

Several observational and randomized studies have been conducted. The RIVAL (RadIal vs. femorAL access for coronary intervention) trial was the first trial aimed to compare radial and femoral access in 7021 patients with acute coronary syndrome (ACS) undergoing PCI. In this cohort, a significant reduction in local vascular complications (hematoma and pseudoaneurysm needing closure) was observed, whereas no differences were noted for ischemic events [37]. Subsequently, the MATRIX (Minimizing Adverse Haemorrhagic Events by TRansradial Access Site and Systemic Implementation of angioX) trial showed a significant reduction in major bleeding and all-cause mortality in 8404 patients with ACS undergoing PCI [38]. Yet, the latter trial also showed a significant reduction in acute kidney injury with TRA compared with TFA [39]. Other studies have consistently demonstrated the lower rates of bleeding associated with TRA compared with TFA; however, the potential benefit of TRA on mortality was uncertain given the contrasting results of trials and the fact that none of them could be properly powered for such an endpoint. Therefore, a recent individual patient data meta-analysis of seven randomized clinical trials including 21,600 patients undergoing coronary angiography with or without PCI that compared TRA versus TFA has been conducted. In this wide cohort, TRA access was associated with lower all-cause mortality and major bleeding at 30 days compared with TFA [40]. The benefit of TRA occurred early (i.e., within 10 days) after PCI and was maintained up to 30 days. Notably, TRA was confirmed to be associated with reduced major bleeding; however, for the first time, the relationship between bleeding and mortality reduction was assessed showing that the bleeding prevention only partially mediated the TRA mortality benefit over TFA. Thus, this meta-analysis provided definitive evidence that TRA should be considered the preferable access site for percutaneous coronary procedures, supporting most recent recommendations on the preferential use of the radial approach [40]. Of course, in some cases, there is failure of TRA, and TFA is needed [41]; however, both TRA should be always prioritized over TFA. Yet, when TFA is needed, every attempt to optimize it in order to reduce complications should be considered (i.e., ultrasound-guided TFA, adequate compression, or closure device utilization) [42,43,44].

### 2.3. Stent Choice

Over the last decades, devices used during PCI advanced from bare metal stents (BMS) to DES that employ the elution of an antiproliferative drug within the vessel wall to reduce neointimal proliferation and in-stent restenosis. The advent of newer-generation DES, displaying thinner struts and more biocompatible polymers, was associated with reduced thrombogenicity and ST rates, even lower than those observed with BMS [45]. Therefore, the reduced need for antithrombotic protection shifted the research to DAPT modulation strategies aimed at mitigating the post-PCI bleeding risk [19,20,21,46].

DES and BMS in HBR patients receiving short DAPT were compared in few randomized trials. In the LEADERS FREE (A Prospective Randomized Comparison of the BioFreedom Biolimus A9 Drug Coated Stent Versus the Gazelle Bare Metal Stent in Patients With High Risk of Bleeding) trial, 2466 patients with either ACS or chronic coronary syndrome (CCS) at HBR were randomized to receive either the BioFreedom polymer-free Biolimus-coated stent or the Gazelle BMS prior to receiving 1 month of DAPT. The primary endpoint, a composite of cardiac death, myocardial infarction (MI), or ST, was significantly lower with the BioFreedom DES [47]. In the ZEUS (Zotarolimus-eluting Endeavor Sprint Stent in Uncertain DES Candidates) trial, 1606 patients with stable or unstable symptoms, qualified as uncertain candidates for DES, were randomized to receive the Endeavor stent, a hydrophilic polymer-based, second-generation zotarolimus-eluting stent (ZES) with a unique drug fast-release profile, or BMS, and all received a 1-month DAPT. Among uncertain candidates, there were 828 patients fulfilling pre-defined clinical or biochemical HBR criteria, including advanced age, indication for oral anticoagulants or other pro-hemorrhagic medications, history of bleeding, and known anemia. HBR patients had worse outcomes, owing to higher ischemic and bleeding risks compared with those non-HBR, and most of all, in these patients ZES was associated with significantly lower rates of major adverse cardiovascular events (MACEs), MI, target vessel revascularization (TVR), and ST [48,49,50]. The SENIOR (Efficacy and Safety of New Generation Drug Eluting Stents Associated With an Ultra Short Duration of Dual Antiplatelet Therapy. Design of the Short Duration of Dual antiplatElet Therapy With SyNergy II Stent in Patients Older Than 75 Years Undergoing Percutaneous Coronary Revascularization) trial enrolled 1200 patients aged 75 years or older presenting with ACS or CCS. Participants were randomized to receive either the Synergy bioabsorbable polymer everolimus-eluting DES or a thin strut BMS (i.e., Omega or Rebel), followed by a short DAPT duration (6 months for ACS and 1 month for CCS) in both groups. Synergy use was associated with a lower risk of 1-year composite of all-cause mortality, MI, stroke, or ischemia-driven target lesion revascularization (TLR) [51].

With the endorsement of DES for use in HBR, stent manufacturers mainly focused on proving the noninferiority of their own devices to DES already labeled for this indication. In the ONYX ONE (A Randomized Controlled Trial With Resolute Onyx in One Month Dual Antiplatelet Therapy for High-Bleeding Risk Patients) trial, the Resolute Onyx durable-polymer zotarolimus-eluting stent was randomly compared to the BioFreedom DES in 1996 ACS or CCS patients with HBR features (mainly represented by older age and the need for anticoagulation) receiving one-month DAPT. Noninferiority was met in the composite of cardiac death, MI, or ST at 1 year [52].

Furthermore, the Bioflow-DAPT trial randomized 1948 patients at HBR (according to the Academic Research Consortium for HBR [ARC-HBR] definition or to a high PRECISE-DAPT score) presenting with ACS or CCS to receive either the Orsiro Mission biodegradable polymer sirolimus-eluting stent or the Resolute Onyx stent, both followed by one-month DAPT. Orsiro Mission was noninferior to Resolute Onyx in terms of the composite of cardiac death, MI, or ST at 1 year [53]. Several registries or trials have also been conducted or are under investigation to explore safety and efficacy of other DES in HBR patients.

The case of the Ultimaster biodegradable polymer sirolimus-eluting stent is unique because the MASTER DAPT (Management of High Bleeding Risk Patients Post Bioresorbable Polymer Coated Stent Implantation With an Abbreviated Versus Prolonged DAPT Regimen) trial is the only dedicated trial to explore a short DAPT in HBR patients. Specifically, it compared abbreviated (one month) and standard DAPT (at least three months) in patients with HBR features undergoing PCI with implantation of the Ultimaster biodegradable polymer sirolimus-eluting stent. The randomization occurred at 1 month from PCI. Short DAPT was noninferior to standard DAPT in terms of net adverse cardiovascular events (NACEs) and MACEs, but was superior in reducing the rates of major or clinically relevant nonmajor bleeding (CRNMB) [54].

Regardless of stent type, the length and the diameter of the single stent also matters. In a cohort of 3145 consecutive patients undergoing DES implantation, the length of the stented segment was independently associated with the incidence of ST and death or MI after PCI. The value of stent length ≥ 31.5 mm was a threshold for the prediction of ST [55]. Furthermore, pooled angiographic and intracoronary imaging data from 14 trials with DES implantation showed that small vessels treated with smaller stents were associated with greater adverse events [56], even more in female patients who more often have small vessels [57].

On this background, the optimal stent choice and its implantation is pivotal to optimize outcomes in patients undergoing PCI, and the use of intracoronary imaging could be useful to achieve optimal results in terms of stent sizing.

### 2.4. Intracoronary Imaging

Intracoronary imaging with both intravascular ultrasound (IVUS) or optical coherence tomography (OCT) allows lesion characteristic assessment, precise stent sizing, and stent implantation evaluation with the aim of optimize the PCI results [58]. Although both IVUS and OCT can provide detailed images of the coronary artery wall, lumen, and stent structure, there are some differences between these tools. OCT has the advantage of 10 times the resolution of IVUS, allowing for a more accurate assessment of plaque composition, but with a shallower image and a greater use of contrast medium. In contrast, IVUS can hardly evaluate vessel wall components, but it enables a complete visualization of the entire vessel cross-section (i.e., plaque burden) and ostium segments and does not require contrast medium. As they are equally capable of assessing vessel lumen, both IVUS and OCT allow the evaluation of minimum stent area (MSA) in order to detect stent under-expansion, which is the most important feature of suboptimal stent implantation [59]. Stent under-expansion is a well-established major predictor of stent failure and, therefore, of increased risk of adverse cardiovascular events [60,61]. Suboptimal stent implantation is defined by a relative stent under-expansion of <80% or absolute MSA of <4.5 mm^2^ at OCT and <5.5 mm^2^ at IVUS [58].

Intracoronary imaging use has been associated with a significant improvement in PCI results and, thereby, with better clinical outcomes.

The first large randomized trial evaluating IVUS-guided PCI was the IVUS-XPL (Impact of Intravascular Ultrasound Guidance on Outcomes of Xience Prime Stents in Long Lesions) trial enrolling 1400 patients with long coronary lesions (implanted stent length ≥ 28 mm). In this cohort, IVUS-guided PCI was superior to angiography-guided PCI in reducing target vessel failure (TVF), a composite of cardiac death, target lesion MI, or ischemia-driven TLR at 5 years, mainly driven by a lower risk of TLR [62].

This finding was confirmed by the more recent ULTIMATE (Intravascular Ultrasound Guided Drug Eluting Stents Implantation in ‘All-Comers’ Coronary Lesions) trial, including 1448 all-comer patients and comparing IVUS vs. angiographic guidance for DES implantation. At 3 years, IVUS-guided DES implantation was associated with significantly lower rates of TVF and ST, especially in patients who underwent an IVUS-defined optimal procedure [63].

As far as OCT is concerned, the first large randomized trial was the OCTOBER (OCT or Angiography Guidance for PCI in Complex Bifurcation Lesions) trial, enrolling 1201 patients with complex bifurcation lesions. OCT-guided PCI was associated with a lower incidence of MACEs (a composite of death from cardiac causes, target lesion MI, or ischemia-driven TLR) at 2 years compared with angiography-guided PCI [64]. These results were corroborated in the ILLUMIEN-IV (OCT-guided coronary stent implantation compared with angiography) trial, a randomized study enrolling 2487 patients with medically treated diabetes mellitus or complex coronary artery lesions. At 2-year follow-up, the rate of TVF (a composite of death from cardiac causes, target vessel MI, or ischemia-driven TVR) was not significantly different between the groups. However, the final MSA was larger in the OCT group compared with angiography guidance, resulting in a significantly lower incidence of ST [65].

Finally, the RENOVATE-COMPLEX PCI (Randomized Controlled Trial of Intravascular Imaging Guidance vs. Angiography-Guidance on Clinical Outcomes after Complex Percutaneous Coronary Intervention) trial enrolled 1639 patients with complex coronary-artery lesions, such as true bifurcations, chronic total occlusion, unprotected left main, long coronary lesions (implanted stent ≥ 38 mm in length), and severely calcified lesions. In the intravascular imaging-guided PCI group, the choice of IVUS or OCT was left to the operator discretion. At the 2-year follow-up, intracoronary imaging guidance was associated with a significantly lower rate of TVF (composite of death from cardiac causes, target vessel–related MI, or clinically driven TVR), and the pre-specified subgroup analysis showed a consistent benefit regardless of used imaging modality. Noteworthy, this was the first trial showing a significant reduction in cardiac mortality [66].

More recently, in a large meta-analysis including nearly 16,000 patients from 22 randomized clinical trials, imaging-guided PCI reduced the risk of death, MI, repeat revascularization, and ST compared with angiography-guided PCI [67]. In another large meta-analysis, imaging-guided PCI was associated with a reduction in ischemia-driven target lesion revascularization compared with angiography-guided PCI, with the difference most evident for IVUS, but no significant differences in MI were observed between guidance strategies [68].

Recently, Lee et al., combining data from the RENOVATE-COMPLEX-PCI randomized trial (n = 1639) and a registry from a single institution (n = 2972), used a complexity score that integrates nine factors of lesion complexity. They showed that a complexity score ≥ 3 independently predicted TVF and that intracoronary imaging conferred an increasing benefit with increasing PCI complexity. Notably, imaging-guided PCI showed a lower risk of TVF across all degrees of lesion complexity [69].

These data suggest that the greater the complexity of PCI is, the more the benefit obtained with the intracoronary imaging use. Certainly, long coronary lesions, heavily calcified lesions, and left main involvement and true bifurcation lesions require a more careful approach that can be achieved solely with intracoronary imaging assistance.

Intracoronary imaging use could have a significant role also in the choice of antithrombotic therapy. Indeed, in the IVUS-XPL trial, the 1400 patients undergoing PCI were randomized in a 2 *×* 2 factorial design to IVUS-guided vs. angiography-guided PCI and subsequently to 6-month vs. 12-month DAPT with aspirin and clopidogrel. In this cohort, a significant interaction was observed for IVUS use and the duration of DAPT with better clinical outcomes with prolonged DAPT in patients undergoing angiography-guided PCI, while a greater benefit was found with short DAPT in patients undergoing IVUS-guided PCI. This result suggests that IVUS-guided PCI with stent optimization may enhance the safety of a shorter DAPT [62]. This could be one of the reasons for the successful results of ticagrelor monotherapy in the ULTIMATE-DAPT (Ticagrelor alone versus ticagrelor plus aspirin from month 1 to month 12 after percutaneous coronary intervention in patients with acute coronary syndromes) trial that enrolled patients with ACS previously included in the IVUS-ACS (Intravascular ultrasound-guided versus angiography-guided percutaneous coronary intervention in acute coronary syndromes) study. In this trial, after 1-month DAPT, ticagrelor monotherapy was associated with significant reduction in clinically relevant bleeding with similar rate of major adverse cardiovascular and cerebral events (MACCEs), compared with DAPT of aspirin plus ticagrelor. Remarkably, although there was no interaction between IVUS use and DAPT duration in terms of MACCE rates, the crude numbers indicate that in ticagrelor monotherapy group, IVUS-guided PCI was associated with a lower MACCE rate, compared with angiography-guided PCI, suggesting the potential benefits of intracoronary imaging [70].

An ongoing randomized trial (SHORTDAPT IVUS trial) is enrolling 3566 patients to assess 1-year efficacy and safety of shortening DAPT duration with IVUS guidance in PCI patients (clinicaltrials.gov identifier: NCT06648720).

Therefore, it could be argued that the achievement of better procedural results with increasing use of intracoronary imaging might allow for safe DAPT or dual antithrombotic therapy (DAT) shortening strategies, even in patients with AF undergoing PCI, with the aim of reducing bleeding risk.

## 3. European and American Guideline Recommendations About Procedural Strategies

Both most recent European and American guidelines strongly recommend radial access as the standard approach for coronary angiography and PCI in both acute and elective settings (Class I, Level of Evidence A). However, femoral access could be chosen instead of radial access in some patients, depending on the hemodynamic status and other technical aspects during the PCI procedure [24,32,71].

No specific recommendations are provided by the European and American guidelines about the stent choice, except for the preference of DES over BMS in all patients undergoing PCI from the American guidelines (Class I, Level of Evidence A) [32] and in all ACS patients for the ESC guidelines [24]. Furthermore, the European guidelines strongly recommend the preference of DES over drug-coated balloons for the treatment of in-DES restenosis (Class I, Level of Evidence A) [71].

Regarding intracoronary imaging, the European ACS guidelines state that it should be considered to guide PCI (Class IIa, Level of Evidence A) [24], whereas the CCS guidelines strongly recommend intracoronary imaging guidance using IVUS or OCT when performing PCI on anatomically complex lesions, in particular left main stem, true bifurcations, and long lesions (Class I, Level of Evidence A) [71].

Consistently, the most recent guidelines from the American College of Cardiology (ACC)/American Heart Association (AHA) for coronary artery revascularization suggest the use of IVUS for procedural guidance in patients undergoing coronary stent implantation, particularly for left main stenting or for complex PCI (Class IIa, Level of Evidence B). OCT is considered as a reasonable alternative to IVUS, except for ostial left main lesions (Class IIa, Level of Evidence B). Furthermore, for patients with stent failure, IVUS or OCT could be used to determine the mechanism of stent failure [32].

## 4. Antithrombotic Strategies

Patients with AF undergoing PCI are peculiar given their thrombotic and bleeding risks. To date, most evidence, and therefore international guidelines, support that these patients need a certain period of triple antithrombotic therapy (TAT), that is the combination of DAPT and OACs, to mitigate coronary ischemic risks and to reduce stroke and systemic embolism. Nevertheless, TAT is associated with a greater risk of bleeding. Yet, the bleeding risk of these patients can be further increased by the presence of additional HBR criteria [7,72,73,74], with an effect that is entirely consistent regardless of sex and clinical presentation [75,76]. Consequently, over time, some alternative strategies, such as short TAT durations, have been tested. Currently, there are also studies exploring the omission of the OAC in the first period after PCI [7,77].

### 4.1. Assessment of Ischemic and Bleeding Risks

AF is a leading cause of stroke and thromboembolism and is preventable using OACs. The current European guidelines recommend OACs in all patients with clinical AF at elevated thromboembolic risk in order to prevent ischemic stroke and thromboembolism [78]. The CHA2DS2–VASc score is the most used one. It is a simple clinical score giving points for congestive heart failure, hypertension, age ≥75 years (2 points), diabetes mellitus, prior stroke/TIA/thromboembolism (2 points), vascular disease, age 65–74 years, and female sex. It is the most used risk score across Europe. However, since female sex is only an age-dependent stroke risk modifier, the current evidence suggests using CHA2DS2–VA. OACs are recommended in patients with a score of 2 or more (Class I, Level of Evidence C) and should be considered in patients with a score of 1 (Class IIa, Level of Evidence C). Nevertheless, the main limitation is the lack of other parameters with an established association with thromboembolism, such as cancer, chronic kidney disease (CKD), ethnicity, and biomarkers including troponin and B-type natriuretic peptide. Atrial enlargement, hyperlipidemia, smoking, and obesity also emerged as additional risk factors in some studies. Patients undergoing PCI have specific thrombotic risks related to several factors. The 2023 ESC guidelines on ACS defined high ischemic risk by the presence of clinical characteristics (i.e., chronic kidney disease, prior ST on antiplatelet therapy), procedural features (i.e., at least three stents implanted, at least three lesions treated, bifurcation with two stents implanted, stenting of the last remaining patent coronary artery, total stent length >60 mm, treatment of a chronic total occlusion) and coronary anatomy (i.e., multivessel disease, complex coronary lesions) [78]. Nevertheless, it is unclear how these criteria should guide the selection of an antithrombotic regimen in AF-PCI patients. Data from trials did not identify specific subgroups of patients in whom TAT should be considered due to a significant benefit described, although pooled data from trials show that decision making should be individualized on the baseline risk of thrombosis and bleeding [17].

Several scores of bleeding have been developed for patients with AF, but its role is still unclear. While 2020 ESC guidelines on AF suggested a formal risk score-based assessment of bleeding risk using the HAS-BLED and a strict follow up for patients with HAS-BLED > 3 [79], the most recent ones in 2024 did not refer to the score recommending a generic assessment and management of modifiable bleeding risk factors in all patients eligible for OACs, and specifically suggesting against prescription and withdrawal of OACs based on specific risk scores. Prescription of proton pump inhibitors (PPIs) is common in patients taking OACs, although the evidence is limited and not specifically in AF patients. Thus, guidelines do not include a dedicated recommendation and suggest individualizing the decision based on the overall perceived bleeding risk [78].

In the setting of patients undergoing PCI, the use of the PRECISE-DAPT score and ARC-HBR criteria are recommended to assess the bleeding risk. However, these tools may be discordant in some patients who have a greater risk of bleeding compared with those in whom both are at low risk, as shown recently in a large population from multiple studies [16]. For this reason, the PRECISE-HBR score was generated and validated. It is a simple seven-item (age, estimated glomerular filtration rate, hemoglobin, white blood cell count, previous bleeding, oral anticoagulation, and ARC-HBR criteria) risk score to predict bleeding after PCI that demonstrated a moderate improvement in discrimination over other available scores. Specifically, the score was derived in a cohort of 29,188 patients undergoing PCI, of whom 1136 (3.9%) had bleeding ARC (BARC) type 3 or 5 at 1 year. The data were obtained from four contemporary real-world registries and the XIENCE V USA trial and then were externally validated in 4578 patients from the MASTER DAPT trial and 5970 patients from the STOPDAPT-2 total cohort. Notably, this study confirmed the intuitive concept and previous evidence that having more HBR factors is associated with increasing bleeding risk. It also introduced the new category of very HBR defined by a 1-year risk of BARC type 3 or 5 bleeding of ≥6% [16]. The PRECISE-HBR could be a useful tool to identify patients who require a short course of triple antithrombotic therapy after PCI and could guide an earlier OAC monotherapy strategy after a shorter dual antithrombotic regimen, but future investigations will be needed.

Notably, the assessment and monitoring of bleeding and ischemic risks should be dynamic over time considering their variability related to changes in risk factors or to acute events during follow-up (e.g., ACS, major bleeding events, increased age, worsening renal function, etc.). In these scenarios, drugs withdrawal or escalation or dose adjustment for OACs could be required over time. A careful assessment is particularly required for elderly patients with multiple comorbidities. This special and fragile cohort is at high risk of both bleeding and thrombotic events. Yet, in these patients, reduced doses of OACs are pivotal to mitigate the bleeding risk. Dose reduction is primarily recommended for specific features that increase bleeding risk (CKD, low body weight, and very elderly patients) [80]. However, reduced-dose direct OACs (DOACs) are commonly prescribed also to older patients with multiple comorbidities who are at high bleeding risk, even in the absence of the dose-reduction criteria. This strategy showed good efficacy in preventing stroke or systemic embolism without increasing the bleeding risk in this population [81,82], but further evidence is needed before recommending reduced doses of OACs out of the dose-reduction criteria.

### 4.2. Evidence from Registries, Trials, and Meta-Analyses

Randomized trials are the ideal way to provide results to drive evidence-based medicine. However, observational studies remain relevant to assess real-world experience from large and unselected populations, to deliver a snapshot on how patients are managed, and to offer alternative hypotheses compared to randomized trials. Different large registries have been published in the setting of AF or OAC patients undergoing PCI. In the AVIATOR-2 registry, 514 nonvalvular AF-PCI patients were included to assess the antithrombotic therapy patterns, agreement between subjective physician ratings and validated risk scores, physician–patient perceptions influencing antithrombotic therapy, and 1-year outcomes [83]. TAT was prescribed in 66.5%, DAPT in 20.7%, and DAT in 12.8% of patients, but there were no differences in 1-year outcomes. In this digital health study, there was poor agreement between physician ratings and validated risk scores, as well as for risk perception with physicians rating safety first when prescribing while patients feared stroke over bleeding. In the WOEST-2 (dual versus triple antithrombotic therapy after percutaneous coronary intervention) study, 1075 patients on OACs undergoing PCI were prospectively enrolled between 2014 and 2021 [84]. Most indications for OACs were AF (93.6%) and mechanical heart valve prosthesis (4.7%). DOACs were prescribed in 53.1% and vitamin-K antagonists (VKAs) in 46.9% of patients. At discharge, 60.9% received DAT, and 39.1% received TAT. The comparison of DAT (P2Y12 inhibitor and OAC) vs. TAT (aspirin, P2Y12 inhibitor, and OAC) at 1 year showed that DAT was associated with less clinically relevant and similar major bleeding events with a numerical, but not statistical significant, increase in ischemic events [84]. In the MATADOR-PCI (Management of Antithrombotic TherApy in Patients with Chronic or DevelOping AtRial Fibrillation During Hospitalization for PCI) prospective registry, 598 consecutive patients with a confirmed diagnosis of ACS (46% STEMI) and concomitant AF (pre-existent or new onset) undergoing PCI were analyzed. TAT was still largely prescribed, predominantly associated with low dosages of DOACs and clopidogrel and for <6 months after PCI [85]. In the recent PERSEO (PERcutaneous coronary interventions in Patients Treated with Oral Anticoagulant Therapy) study, 1234 consecutive patients with an indication for OACs and undergoing PCI were included from 2018 to 2022. The main indications for OACs were AF (86%), followed by ventricular thrombosis (5%) and venous thrombosis (2.3%). Of the 1228 patients discharged alive, 222 (18%) were on VKAs, and 1006 (82%) were on DOAC (*p* < 0.01). DAT was prescribed in 197 patients, whereas TAT was prescribed in 1028. At the 1-year follow-up, the NACE rate was significantly higher with VKAs compared to DOAC (23% vs. 16%, *p* = 0.013). DAT was associated with lower bleeding rates with no differences in the NACE rate [9].

The first trial to investigate a DAT strategy after PCI was the WOEST (What is the Optimal antiplatElet and Anticoagulant Therapy in Patients With Oral Anticoagulation and Coronary StenTing) trial in which the innovative hypothesis to interrupt aspirin was tested. Specifically, the investigators hypothesized that clopidogrel was essential to prevent ST, while aspirin withdrawal would reduce the bleeding risk. The trial assessed a DAT regimen based on OAC plus clopidogrel compared with standard TAT with OAC plus DAPT. With an open-label design, 573 patients on OACs undergoing PCI were randomized to DAT or TAT, and bleeding events within 1 year were significantly reduced without an apparent increase (rather a numerical decrease) in thrombotic events, such as MI or ST [86]. This strategy has guided subsequent trials that have never assessed clopidogrel interruption with aspirin prolongation. Despite being pivotal, this trial had peculiar aspects to be accounted for when interpreting its findings. First, 69% of patients were AF-PCI, while 31% had different indications for OACs, including mechanical valves, apical aneurysm, and others. Second, 67% of patients received TAT for 1 year. Third, the DAT group showed a nominally significant lower rate of all-cause death without a clear explanation that has been matter of discussion for years. Subsequently, the ISAR-TRIPLE (Intracoronary Stenting and Antithrombotic Regimen-Testing of a 6-Week Versus a 6-Month Clopidogrel Treatment Regimen in Patients With Concomitant Aspirin and Oral Anticoagulant Therapy Following Drug-Eluting Stenting) trial investigated whether shortening the duration of clopidogrel from 6 months to 6 weeks after PCI with a DES was superior in terms of NACEs of death, MI, definite ST, stroke, or major bleeding at 9 months in patients receiving concomitant aspirin and OAC. In total, 614 patients were randomized to the 2 TAT regimens, but no significant differences emerged for all outcomes including the primary NACE outcome, the secondary outcome of the combined ischemic endpoint (cardiac death, MI, ST, and ischemic stroke), or for the secondary bleeding endpoint (thrombolysis in myocardial infarction [TIMI] major bleeding) [87].

After the advent of large randomized trials demonstrating noninferiority and/or superiority of DOACs versus VKAs in the setting of nonvalvular AF, these drugs have been recommended over VKAs and widely used. Consequently, also in the specific subset of patients with AF undergoing PCI, four trials have been conducted on the four DOACs (Table 1). In the PIONEER-AF PCI (Open-Label, Randomized, Controlled, Multicenter Study Exploring Two Treatment Strategies of Rivaroxaban and a Dose-Adjusted Oral Vitamin K Antagonist Treatment Strategy in Subjects with Atrial Fibrillation who Undergo Percutaneous Coronary Intervention) trial, 2124 AF patients undergoing PCI were randomly assigned to three groups: (1) 15 mg rivaroxaban once daily plus a P2Y12 inhibitor for 12 months; (2) 2.5 mg rivaroxaban twice daily plus DAPT for 1, 6, or 12 months; or (3) VKAs plus DAPT for 1, 6, or 12 months. Compared with VKAs, rivaroxaban (pooling the 2 dose arms) was associated with lower rates of clinically significant bleeding with similar rates of cardiovascular death, MI, or stroke. A non-significant greater risk of stroke and ST was observed in both rivaroxaban arms, but it is important to highlight that these two doses were lower than the 20 mg investigated and approved for stroke prevention in AF [88]. In the RE-DUAL PCI (Randomized Evaluation of Dual Antithrombotic Therapy with Dabigatran versus Triple Therapy with Warfarin in Patients with Nonvalvular Atrial Fibrillation Undergoing Percutaneous Coronary Intervention) trial, 2725 AF patients undergoing PCI were randomized to TAT with VKAs plus a P2Y12 inhibitor (clopidogrel or ticagrelor) and aspirin for 1–3 months or DAT with dabigatran (110 mg or 150 mg twice daily) plus a P2Y12 inhibitor (clopidogrel or ticagrelor). At 14 months, the low dose was associated with lower rate of the primary endpoint of major or CRNMB events (15.4% in the 110 mg DAT group vs. 26.9% in TAT group; HR: 0.52; 95% CI: 0.42 to 0.63; *p* < 0.001 for noninferiority; *p* < 0.001 for superiority), while the high dose was noninferior (20.2% in the 150 mg DAT group vs. 25.7% in TAT group; HR: 0.72; 95% CI: 0.58–0.88; *p* < 0.001 for noninferiority). Both dabigatran arms were noninferior to TAT for the composite of death, MI, stroke, systemic embolism, or unplanned revascularization, but a numerically higher risk of ST was found in the 110 mg group compared with TAT [89]. There were consistent findings when patients were stratified by clinical presentation [90]. In the ENTRUST-AF PCI (Edoxaban Treatment Versus Vitamin K Antagonist in Patients With Atrial Fibrillation Undergoing Percutaneous Coronary Intervention) trial, 1506 patients were randomized to 60 mg edoxaban once daily plus a P2Y12 inhibitor for 12 months or TAT with a VKA, a P2Y12 inhibitor, and aspirin (for 1 to 12 months) [91]. Noninferiority was demonstrated, but superiority of DAT with edoxaban compared with VKA-TAT was not observed for the 1-year major or CRNMB rate (HR: 0.83; 95% CI: 0.65–1.05; *p* = 0.001 for noninferiority and *p* = 0.115 for superiority), without any difference in the composite efficacy outcome. Nevertheless, a post-hoc analysis with a landmark at 14 days for the primary bleeding outcome showed a clear signal of heterogeneity (*p* for interaction < 0.0001) with a non-significantly lower bleeding rate for VKA-TAT vs. edoxaban-DAT in the early period (hypothesized to be related to subtherapeutic international normalized ratio [INR]) but with a subsequent superiority of edoxaban-DAT with significant lower bleedings (HR for edoxaban 0.68, 95% CI 0.53–0.88) [92]. In the AUGUSTUS (An Open-label, 2-by-2 Factorial, Randomized Controlled, Clinical Trial to Evaluate the Safety of Apixaban vs. Vitamin K Antagonist and Aspirin vs. Placebo in Patients with Atrial Fibrillation and Acute Coronary Syndrome and/or Percutaneous Coronary Intervention) trial, 4614 AF patients with ACS or CCS undergoing PCI or medically managed were randomized with a 2 × 2 factorial design to four groups: either 5 mg apixaban twice daily or a VKA (open label) and to either aspirin or placebo (blinded) for 6 months [93]. No significant interaction emerged between the two randomization factors for all endpoints. Apixaban was associated with lower rate of major or CRNMB events and the composite of death or hospitalization, in the absence of differences in the composite ischemic endpoint compared with VKAs. On the other hand, aspirin was associated with a higher risk of major and CRNMB events compared with placebo (16.1% vs. 9.0%), but similar risk of death or hospitalization and ischemic events compared with placebo. However, a numerically higher rate of ST was observed with aspirin discontinuation. ST was not frequent, mostly occurring early after PCI and was lower with aspirin compared with placebo and with apixaban compared with VKAs [94]. In another sub-analysis, aspirin immediately and for up to 30 days was associated with an equal tradeoff between higher severe bleeding and lower severe ischemic events. After 30 days, aspirin increased bleeding without significantly reducing ischemic events [95].

These trials provided fundamental evidence on the peri-procedural management of these patients. However, some aspects should be taken into account. First, these four trials were powered for demonstrating the superiority of DAT vs. TAT in reducing bleeding, but their sample size was inadequate to properly evaluate major ischemic events or rare bleeding events (i.e., intracranial bleeding). Second, the main comparison was DOAC-based DAT vs. VKA-based TAT except for the AUGUSTUS trial. Third, aspirin was administered in the peri-PCI period; thus, DAT patients also received a short period of TAT (in PIONEER AF-PCI, REDUAL-PCI, and ENTRUST-AF-PCI, aspirin was used on average for 1–2 days after PCI; in AUGUSTUS, it was used for 6 days). Fourth, the specific duration of TAT and the type of P2Y12 inhibitor need to be further investigated. Finally, the results of patients with high ischemic risk or with ACS at presentation are derived from subgroup analyses. On this background, meta-analyses may provide additional information, increasing the power of analyses and providing comprehensive evidence on the topic. The four DOAC trials were firstly pooled in the meta-analysis included in the ENTRUST-AF PCI publication. Compared with VKA-TAT (n = 3585), DOAC-DAT (n = 4342) was associated with lower risks of major or CRNM bleeding events and similar risks of MACEs, all-cause death, and stroke, with numerical increases in MI and ST [91]. A successive more detailed meta-analysis included all 10,234 patients from these trials to compare DAT vs. TAT (DAT = 5496 vs. TAT = 4738) [17]. Overall, DAT was associated with significant lower rates of the primary safety endpoint (International Society of Thrombosis and Hemostasis [ISTH] major or CRNMB) compared with TAT (13.4% vs. 20.8%; RR: 0.66, 95% CI: 0.56–0.78; *p* < 0.0001; I^2^ = 69%), which was guided by reductions in both major and CRNMB events. Conversely, there was a significantly higher risk of ST (1.0% vs. 0.6%; RR: 1.59, 95% CI: 1.01–2.50; *p* = 0.04; I^2^ = 0%) and a borderline higher risk of MI (3.6% vs. 3.0%; RR: 1.22, 95% CI: 0.99–1.52; *p* = 0.07; I^2^ = 0%) with DAT compared with TAT. These findings were consistent when the analysis was restricted to DOAC-based DAT vs. VKA-based TAT. Notably, for the first time, DAT was associated with significant reduction in intracranial hemorrhage (RR 0.33, 95% CI 0.17–0.65; *p* = 0.001; I^2^ = 0%).

ACS has often emerged and been perceived as associated with a greater risk of thrombotic complications, thus influencing the decision making on type and duration of antithrombotic therapy [17]. In this respect, sub-analyses of the PIONEER-AF PCI, RE-DUAL AUGUSTUS, and ENTRUST-AF PCI trials showed that the superior safety and similar efficacy of DAT were consistent across subgroups, including the ACS presentation [92,96,97,98]. Subsequently, a further meta-analysis of all four DOAC-based RCTs focused on assessing the benefits and risks of DAT vs. TAT based on clinical presentation (patients with or without ACS) [18]. Among the 10,193 patients, 5675 presented with ACS (DAT = 3063 vs. TAT = 2612) and 4518 with CCS (DAT = 2421 vs. TAT = 2097). DAT was associated with significant lower bleeding regardless of clinical presentation, including intracranial hemorrhage. The greater rates of MI and ST with DAT vs. TAT were also consistent in ACS and CCS (*p*-int = 0.60 and 0.86, respectively) as was the absence of difference in terms of all-cause death, MACEs, or stroke. These findings suggested that in AF-PCI patients, clinical presentation should not guide therapy. This finding is likely related to the fact that ACS patients are also characterized by a high risk of major bleeding and that the greater absolute and relative risks of MI or ST associated with DAT compared with TAT were not higher in ACS compared with CCS. These unexpected results might reflect the synergistic role of full-dose DOACs with P2Y12 inhibitor monotherapy for the prevention of coronary thrombotic events. Intriguingly, the greater MI and ST risks of DAT compared with TAT were restricted to patients receiving the low-dose of dabigatran (110 mg bid), but not 150 mg bid dabigatran. Consequently, the clinical presentation per se should not drive a specific decision on antithrombotic therapy in patients using DOACs in FDA-approved stroke prevention regimens but should be considered with other recognized ischemic and bleeding risk factors in decision making regarding optimal secondary prevention antithrombotic regimens.

### 4.3. Long-Term OACs

The randomized trials described above addressed the comparison of DAT and TAT after PCI, but long-term antithrombotic therapy in AF-PCI patients remains debated. Although clinical guidelines provide consistent recommendations to interrupt the long-term antiplatelet agent while continuing with an OAC monotherapy, data from randomized trials on a long-term antithrombotic treatment strategy for these patients are still limited (Table 2). The OAC-ALONE (Optimizing Antithrombotic Care in Patients With AtriaL fibrillatiON and Coronary stEnt) trial was a non-inferior trial comparing OAC (either VKA or DOAC) with DAT (OAC plus aspirin or clopidogrel) as long-term strategies beyond 1 year after PCI [99]. The trial was prematurely stopped because of slow recruitment after enrolling 696 patients in 38 months. Patients recruited were less than one-third of the planned patients, and most patients received VKA as OAC. Accounting for these limitations making the trial inconclusive and out of current practice, it failed to prove the non-inferiority of OAC alone to DAT in terms of the composite of all-cause death, MI, stroke, or systemic embolism at 1 year. In the AFIRE (Atrial Fibrillation and Ischemic Events With Rivaroxaban in Patients With Stable Coronary Artery Disease Study) trial, rivaroxaban alone was compared with rivaroxaban-based DAT in 2236 AF patients beyond 1 year after PCI [100]. The trial was stopped early because of increased mortality in the DAT group. At a median follow-up of 24 months, rivaroxaban monotherapy was non-inferior to DAT in terms of the primary efficacy endpoint, namely, a composite of stroke, systemic embolism, myocardial infarction, unstable angina requiring revascularization, or death from any cause (HR: 0.72; 95% CI: 0.55–0.95; *p* < 0.001 for non-inferiority), while superior in terms of bleedings (HR: 0.59; 95% CI: 0.39–0.89; *p* = 0.01 for superiority). In a secondary not pre-specified analysis, superiority for the primary efficacy endpoint was assessed and demonstrated (*p* value was 0.02). In terms of secondary endpoints, all-cause death was lower in the monotherapy group compared with those receiving combination therapy, and this was due to lower rates of both cardiovascular and non-cardiovascular death. Also, the incidence of the composite endpoint of ischemic cardiovascular events or death as well as net adverse clinical events (composite of all-cause death, myocardial infarction, stroke, or major bleeding) were lower in the monotherapy group. The PRAEDO AF (Prospective RAndomized study of safety outcomes treated with EDOxaban in patients with stable CAD and atrial fibrillation) trial investigated the safety of an edoxaban monotherapy in patients with nonvalvular AF and stable CAD, including over 6 months post-implantation of a third-generation DES and 1 year postimplantation of other stents [101]. Overall, 147 patients from eight institutions in Japan were randomly assigned to edoxaban monotherapy (n = 74) or combination therapy (edoxaban plus clopidogrel, n = 73). Major or clinically significant bleeding occurred in two patients in the monotherapy group and five patients in the combination therapy group (HR, 0.39; 95% CI, 0.08–2.02) in the absence of events of myocardial infarction, stent thrombosis, unstable angina requiring revascularization, ischemic stroke, systemic stroke, or hemorrhagic stroke in both groups. The most recent EPIC-CAD (Edoxaban versus Edoxaban with Anti- platelet Agent in Patients with Atrial Fibrillation and Chronic Stable Coronary Artery Disease) trial randomized 524 patients to edoxaban monotherapy and 516 patients to DAT at 18 sites in South Korea [102]. At 12 months, the primary endpoint (composite of death from any cause, MI, stroke, systemic embolism, unplanned urgent revascularization, and major bleeding or clinically relevant nonmajor bleeding) was significantly lower with edoxaban monotherapy versus DAT (6.8% vs. 16.2%; HR, 0.44; 95% CI, 0.30 to 0.65; *p* < 0.001). Major ischemic events were similar, while major bleeding or clinically relevant nonmajor bleeding were significantly lower in the edoxaban monotherapy group (4.7% vs. 14.2%; HR, 0.34; 95% CI, 0.22 to 0.53). None of these trials was powered to detect differences in cardiac or cerebrovascular ischemic events, and some concerns in practice toward DAT discontinuation still remain. However, a recent meta-analysis of these four trials showed that there were no statistically significant differences between OAC monotherapy vs. OAC plus SAPT in the primary effectiveness outcome (defined as a composite of myocardial infarction, ischemic stroke, systemic embolism, or death) as well as myocardial infarction, stent thrombosis, ischemic stroke, all-cause death, or cardiovascular death, while OAC monotherapy was associated with a lower risk of major bleeding [103]. A more recent meta-analysis of five randomized controlled trials, also including the MASTER-DAPT trial, encompassed 5758 patients receiving OACs and with stabilized CAD. Single antithrombotic therapy (SAT) composed of OAC monotherapy showed significant lower risk of major bleeding events without increased risk of ischemic complications compared with DAT composed of OAC plus antiplatelet agent [104]. Importantly, adding the MASTER-DAPT provided additional value to this meta-analysis because it increased the power of the analysis (1666 additional patients included), which is particularly relevant to provide reassuring data in terms of ischemic and more rare thrombotic complications (i.e., stent thrombosis) as well as extending the generalizability of the findings given the indication for OACs (it included other non-AF indications for OACs such as mechanical valves) and the inclusion of non-Asian patients (conversely the 4 AF-OAC trials only included Asian patients).

Focusing on HBR patients, the MASTER-DAPT trial should be considered [54]. This trial is unique because it focused on antiplatelet therapy in HBR patients, including those on OACs and requiring DAT/TAT. As stated above, 1 month after they had undergone successful PCI with Ultimaster (biodegradable polymer sirolimus-eluting stent) implantation, 4579 HBR patients were randomized to abbreviated or standard therapy. Notably, randomization was stratified by concomitant OAC indication. While patients without an OAC indication changed to a single APT for 11 months (abbreviated regimen) or continued ≥5 months of DAPT and single antiplatelet therapy (SAPT) thereafter (non-abbreviated regimen), those with an OAC indication changed immediately to SAPT for 5 months (abbreviated regimen) or continued ≥2 months of DAPT and SAPT thereafter (non-abbreviated regimen). The three ranked primary outcomes were NACEs (a composite of death from any cause, MI, stroke, or major bleeding), MACCEs (a composite of death from any cause, MI, or stroke), and major or clinically relevant nonmajor bleeding; cumulative incidences were assessed at 335 days. The first two outcomes were assessed for noninferiority in the per-protocol population, and the third outcome was assessed for superiority in the intention-to-treat population. Overall, abbreviated therapy was noninferior to standard therapy in terms of both NACEs and MACEs, but significantly reduced the incidence of major or clinically relevant nonmajor bleeding. Specifically, the MASTER-DAPT OAC analysis focused on outcomes in patients with or without OAC [105]. Among the 4579 HBR patients, 1666 (36.4%) patients had an indication for OAC (848 were assigned to the abbreviated antiplatelet therapy [APT] group and 818 to the non-abbreviated APT group), and 2913 (63.6%) had no indication for OAC. The indication for OAC was AF in 84.2%, and the OAC was a DOAC in 64.9% and a VKA in 33.5%, mainly associated with clopidogrel (approximately 98%). In patients with OAC, the median duration of DAPT since coronary stenting was 33 days in the abbreviated arm and 96 days in the nonabbreviated group. There was an almost twofold higher bleeding rate in the OAC subgroup compared with non-OAC patients (10.6% vs. 6.2%) that could be explained in part by the relative long DAPT regimens in the non-abbreviated APT arm of this subgroup but not for the abbreviated APT arm. Moreover, most patients on OACs were treated with a DOAC (64.9%), but there were no outcome differences between DOACs or VKAs. The greater bleeding rates of patients on OACs further highlights the relevance of defining the optimal APT duration and combination in this subgroup who are already categorized as having a HBR. Although most benefit in bleeding reduction was observed in the subgroup of patients without an OAC indication (significant reduction of bleeding in patients without OACs and numerically lower bleedings in those with OACs), the overall findings of the trial were consistent in subgroups of patients with or without OACs, thus supporting that an abbreviated antiplatelet therapy is as effective as and safer than a standard DAPT regimen. However, it should be noted that while clinical guidelines recommend stopping APT after 6 months in an OAC population (Level of Evidence C), this is poorly explored, and the MASTER-DAPT data on OAC subgroup failed to show a clear benefit of stopping APT after 6 months, probably due to the lack of power in this subgroup analysis and the fact that a considerable proportion of patients were nonadherent to the abbreviated allocated treatment regimen prolonging SAPT after 6 months. Therefore, it remains to be explored whether the absence of an increased risk of ischemic events and reduction of bleeding events relates to this nonadherence. Finally, considering that the majority of ST events occur during the first month after ACS/PCI, an emerging alternative antithrombotic strategy for HBR patients is represented by the use of DAPT or ticagrelor monotherapy in the first month, with the omission of OAC, followed by SAPT and OAC [106]. Two ongoing trials (NCT04436978 and NCT05955365) will provide evidence on the safety and efficacy of this strategy [14].

### 4.4. European and American Guideline Recommendations About Antithrombotic Strategies

Most recent ESC guidelines for the management of ACS recommend TAT for 1 week in patients with an OAC indication undergoing PCI, followed by DAT for 12 months, and then OAC alone (Class I, Level of Evidence A). However, DAT for longer than 1 week and up to 1 month should be considered in patients with high ischemic risk or with anatomical and procedural features that overcome the bleeding risk (Class IIa, Level of Evidence C). On the other hand, in patients with a predominant bleeding risk, discontinuation of DAT after 6 months and continuation of OAC alone may be considered (Class IIb, Level of Evidence B). Furthermore, prasugrel or ticagrelor as part of TAT is not recommended (Class III, Level of Evidence C) [24].

For elective cases, the guidelines recommend TAT for 1 week, continuation of DAT for up to 6 months, and then OAC alone (Class I, Level of Evidence A). However, if ischemic risk is a concern, the TAT strategy should be prolonged to 1 month (Class IIa, Level of Evidence B), and DAT should be extended to 12 months (Class I, Level of Evidence A) [71].

Notably, both the European guidelines support the use of DOACs over VKAs, clopidogrel as the antiplatelet agent of choice in DAT, and reduced doses of rivaroxaban (15 mg/day) or dabigatran (110 mg twice daily) when the bleeding risk is high (Class IIa, Level of Evidence B) [24,71].

The American guidelines show consistent recommendations. Indeed, the American guidelines recommend a TAT strategy for 1 week and up to 1 month in patients with a high risk of ST (Class I, Level of Evidence A), followed by DAT for 12 months (6 months if bleeding risk is a concern) and OAC as long-term therapy 1 year after PCI [6,32,107,108].

To summarize, both the European and American guidelines prioritized the reduction of bleeding risk over ischemic risk in patients receiving OAC [109].

**Figure 2:**
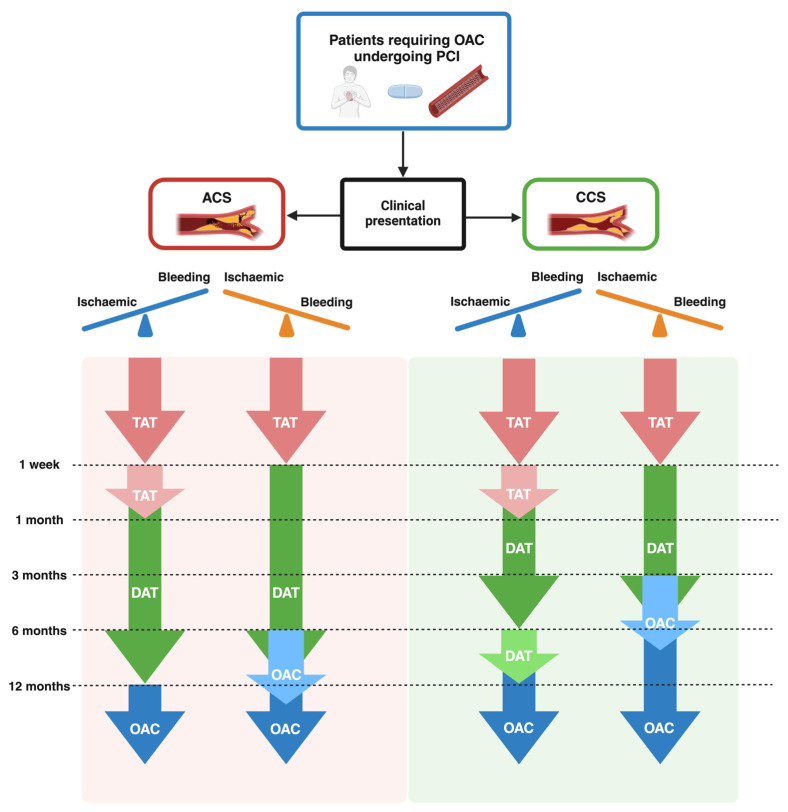
Antithrombotic management in patients requiring OAC undergoing PCI according to the European Society of Cardiology (ESC) and American College of Cardiology (ACC)/American Heart Association (AHA) guidelines. ACS, acute coronary syndrome; CCS, chronic coronary syndrome; DAT, dual antithrombotic therapy; OAC, oral anticoagulation; PCI, percutaneous coronary intervention; SAPT, single antiplatelet therapy; TAT, triple antithrombotic therapy.

The antithrombotic management of patients undergoing PCI and with OAC indication recommended from the guidelines is summarized in Figure 2.

## 5. Conclusions

Patients with AF undergoing PCI represent a special population with significant challenges considering the significant increased risk for bleeding events. For this reason, both peri-procedural and post-procedural strategies to avoid bleeding events are pivotal in this context. The use of radial access, the optimal antithrombotic therapy in the peri-PCI phase, the choice of DES with robust evidence for short antiplatelet therapy, and the use of intracoronary imaging could be helpful strategies to achieve optimal revascularization results, thus minimizing the risks of thrombotic complications after PCI. So far, in the post-PCI phase, the adoption of a short DAT/TAT, the avoidance of drug interactions [110], the dynamic assessment of ischemic/bleeding risk, and the management of co-morbidities could play a key role during the follow-up of these patients. However, further studies are needed to clarify the impact of these strategies, including the optimal type and duration of antithrombotic drugs association (anticoagulant + antiplatelet) and the impact of procedural strategies (such as intracoronary imaging), as well as the impact of risk scores for the optimization of ischemic and bleeding outcomes in patients with AF undergoing PCI. In addition, it is essential to carefully manage these patients by trying to individualize their treatment based on their risks and monitor them over time also considering that their ischemic and bleeding risks might be dynamic during follow-up.

## Figures and Tables

**Table 1:** Main randomized clinical trials comparing ischemic and bleeding outcomes with dual and triple antithrombotic therapy in AF-PCI patients.

TRIAL NAME	WOEST	ISAR-TRIPLE	PIONEER-AF PCI	RE-DUAL PCI	AUGUSTUS	ENTRUST AF-PCI
**IDENTIFIER**	Clinicaltrials.gov NCT0076993	Clinicaltrials.gov NCT00776633	Clinicaltrials.gov NCT01830543	Clinicaltrials.gov NCT02164864	Clinicaltrials.gov NCT02415400	Clinicaltrials.gov NCT02866175
**YEAR**	2013	2015	2016	2017	2019	2019
**PATIENTS (N)**	573	614	2124	2725	4614	1506
**POPULATION**	Patients with OAC indication undergoing PCI (ACS 27.1%)	Patients with OAC indication undergoing PCI (ACS 32.1%)	Patients with AF undergoing PCI with stenting (ACS 51.6%)	Patients with AF undergoing successful PCI (ACS 64.0%)	Patients with AF after ACS, PCI, or both (ACS 61.2%)	Patients with AF undergoing PCI (ACS 52.0%)
**TRIAL DESIGN**	Superiority	Superiority	Superiority	Noninferiority	Noninferiority and superiority	Noninferiority and superiority
**COMPARED TREATMENT STRATEGIES**	OAC + P2Y12I (clopidogrel) for 1 to 12 months vs. OAC + P2Y12I (clopidogrel) + aspirin for 1 to 12 months	OAC + P2Y12I (clopidogrel) + aspirin for 6 weeks vs. OAC + P2Y12I (clopidogrel) + aspirin for 6 months	Rivaroxaban 15 mg qd + P2Y12I (clopidogrel or ticagrelor or prasugrel) for 12 months vs. Rivaroxaban 2.5 mg bid (or 15 mg qd if duration of 1 or 6 months) + DAPT (aspirin and either clopidogrel, ticagrelor, or prasugrel) for 1, 6, or 12 months vs. Warfarin (INR 2.0-3.0) + DAPT (aspirin and either clopidogrel, ticagrelor, or prasugrel)	Dabigatran etexilate 110 mg bid + P2Y12I (clopidogrel or ticagrelor) for 12 months vs. Dabigatran etexilate 150 mg bid + P2Y12I (clopidogrel or ticagrelor) for 12 months vs. Warfarin (INR 2.0-3.0) + DAPT (aspirin for 1 to 3 months and clopidogrel or ticagrelor for 12 months)	Apixaban 5 mg bid + P2Y12I for 6 months vs. Apixaban 5 mg bid + DAPT (aspirin and a P2Y12I) for 6 months vs. Warfarin (INR 2.0-3.0) + P2Y12I for 6 months vs. Warfarin (INR 2.0-3.0) + DAPT (aspirin and a P2Y12I) for 6 months	Edoxaban 60 mg qd + P2Y12I for 12 months vs. VKA + DAPT (aspirin for 1 to 12 months and a P2Y12I for 12 months)
**FOLLOW-UP DURATION**	12 months	9 months	12 months	14 months (mean follow-up)	6 months	12 months
**PRIMARY ENDPOINT**	Any bleeding episode	Composite of death, MI, definite stent thrombosis, stroke, and TIMI major bleeding	Clinically significant bleedings (composite of TIMI major or minor bleeding and bleeding requiring medical attention)	ISTH major or clinically relevant nonmajor bleeding events	ISTH major or clinically relevant nonmajor bleeding events	ISTH major or clinically relevant nonmajor bleeding events
**PRIMARY ENDPOINT RATE**	**19.4% vs. 44.4%** (HR: 0.36, 95% CI: 0.26–0.50, *p* < 0.0001)	**9.8% vs. 8.8%** (HR: 1.14, 95% CI: 0.68–1.91, *p* = 0.63)	**16.8% vs. 18.0% vs. 26.7%** - Group 1 vs. group 3 (HR: 0.59, 95% CI: 0.47–0.76, *p* < 0.001) - Group 2 vs. group 3 (HR: 0.63, 95% CI: 0.50–0.80, *p* < 0.001)	- Group 1 vs. group 3: **15.4% vs. 26.9%** (HR: 0.52, 95% CI: 0.42–0.63, *p* < 0.001) - Group 2 vs. corresponding group 3: **20.2% vs. 25.7%** (HR: 0.72, 95% CI: 0.58–0.88, *p* = 0.002)	- Apixaban vs. VKA: **10.5% vs. 14.7%** (HR: 0.69, 95% CI: 0.58–0.81, *p* < 0.001) - Aspirin vs. placebo: **16.1% vs. 9.0%** (HR: 1.89, 95% CI: 1.59–2.24, *p* < 0.001)	**17.0% vs. 20.0%** (HR: 0.83, 95% CI: 0.65–1.05, *p* = 0.001)
**ANY BLEEDING RATE**	**19.4% vs. 44.4%** (HR: 0.36, 95% CI: 0.26–0.50, *p* < 0.0001)	**37.6% vs. 40.2%** (HR: 0.94, 95% CI: 0.73–1.21, *p* = 0.63)	**16.8% vs. 18.0% vs. 26.7%** - Group 1 vs. group 3 (HR: 0.59, 95% CI: 0.47–0.76, *p* < 0.001) - Group 2 vs. group 3 (HR: 0.63, 95% CI: 0.50–0.80, *p* < 0.001)	- Group 1 vs. group 3: **27.1% vs. 42.9%** (HR: 0.54, 95% CI: 0.46–0.63, *p* < 0.001) - Group 2 vs. corresponding group 3: **33.3% vs. 41.4%** (HR: 0.72, 95% CI: 0.61–0.84, *p* < 0.001)	**7.3% vs. 13.8% vs. 10.9% vs. 18.7%**	**28.0% vs. 32.6%** (HR: 0.84, 95% CI: 0.69–1.01, *p* = 0.06)
**ANY STENT THROMBOSIS RATE**	**1.4% vs. 3.2%** (HR: 0.44, 95% CI: 0.14–1.44, *p* = 0.165)	* 0.7% vs. 0.0% (*p* = 0.50)	**0.7% vs. 0.9% vs. 0.6%** - Group 1 vs. group 3 (HR: 1.20, 95% CI: 0.32–4.45, *p* = 0.79) - Group 2 vs. group 3 (HR: 1.44, 95% CI: 0.40–5.09, *p* = 0.57)	*- Group 1 vs. group 3: **1.5% vs. 0.8%** (HR: 1.86, 95% CI: 0.79–4.40, *p* = 0.15) Group 2 vs. corresponding group 3: **0.9% vs. 0.9%** (HR: 0.99, 95% CI: 0.35–2.81, *p* = 0.98)	**0.91% vs. 0.57% vs. 1.26% vs. 0.69%**	**1.7% vs. 1.3%**
**ALL-CAUSE MORTALITY RATE**	**2.5% vs. 6.3%** (HR: 0.39, 95% CI: 0.16–0.93, *p* = 0.027)	**4.0% vs. 5.2%** (HR: 0.75, 95% CI: 0.35–1.59, *p* = 0.45)	^†^ **2.4% vs. 2.2% vs. 1.9%** - Group 1 vs. group 3 (HR: 1.29, 95% CI: 0.59–2.80, *p* = 0.52) - Group 2 vs. group 3 (HR: 1.19, 95% CI: 0.54–2.62, *p* = 0.66)	- Group 1 vs. group 3: **5.6% vs. 4.9%** (HR: 1.12, 95% CI: 0.76–1.65, *p* = 0.56) Group 2 vs. corresponding group 3: **3.9% vs. 4.6%** (HR: 0.83, 95% CI: 0.51–1.34, *p* = 0.44)	**3.4% vs. 3.3% vs. 3.5% vs. 2.9%**	**6.1% vs. 4.9%** (HR: 1.23, 95% CI: 0.80–1.89, *p* = 0.34)

* In the ISAR TRIPLE and RE-DUAL PCI trials, stent thrombosis was reported as definite stent thrombosis. ^†^ In the PIONEER-AF PCI trial, death was reported as death from cardiovascular causes. Abbreviations: ACS, acute coronary syndrome; AF, atrial fibrillation; BARC, Bleeding Academic Research Consortium; BMS, bare metal stent; CABG, coronary artery bypass graft; CAD, coronary artery disease; DAPT, dual antiplatelet therapy; DES, drug-eluting stent; HBR, high bleeding risk; ISTH, International Society on Thrombosis and Haemostasis; MACCE, major adverse cardiac and cerebrovascular event; MI, myocardial infarction; NACE, net adverse clinical event; OAC, oral anticoagulation therapy; P2Y12I, P2Y12 inhibitors; PCI, percutaneous coronary intervention; SAPT, single antiplatelet therapy; TIMI, Thrombolysis In Myocardial Infarction; VKA, vitamin K antagonist.

**Table 2:** Main randomized clinical trials comparing ischemic and bleeding outcomes with dual antithrombotic therapy and OAC monotherapy for long-term antithrombotic therapy in OAC-PCI patients.

TRIAL NAME	OAC-ALONE	AFIRE	MASTER DAPT (OAC Subgroup with Landmark Data After 6 Months)	PRAEDO-AF	EPIC-CAD
**IDENTIFIER**	Clinicaltrials.gov NCT01962545	Clinicaltrials.gov NCT02642419	Clinicaltrials.gov NCT03023020	Japan Registry of Clinical Trials jRCTs031180119	Clinicaltrials.gov NCT03718559
**YEAR**	2019	2019	2021	2022	2024
**PATIENTS (N)**	690	2215	1666	147	1040
**POPULATION**	Patients with AF and stable CAD who underwent PCI more than 1 year earlier	Patients with AF and stable CAD who underwent PCI or CABG more than 1 year earlier or who had angiographically confirmed CAD not requiring revascularization	HBR patients with an OAC indication who underwent PCI after 1 month of DAPT	Patients with nonvalvular AF and stable CAD 6 months after the implantation of a third-generation DES and 1 year after the implantation of other stents	Patients with nonvalvular AF (prevalent or paroxysmal) with high embolic risk (CHA_2_DS_2_-VASc score ≥ 2) and with stable CAD defined as prior revascularization (PCI or CABG) ≥6 months for chronic CAD and ≥12 months for ACS or anatomically confirmed CAD (≥50% stenosis in CAG or CCTA) on medical therapy alone
**TRIAL DESIGN**	Non-inferiority	Non-inferiority and superiority	Non-inferiority and superiority	Superiority	Superiority
**COMPARED TREATMENT STRATEGIES**	OAC (DOAC or warfarin) monotherapy for 12 months vs. OAC (DOAC or Warfarin) + SAPT (aspirin or clopidogrel) for 12 months	Rivaroxaban 15 mg monotherapy for 24 months vs. Rivaroxaban + SAPT (aspirin or P2Y12I) for 24 months	Abbreviated DAPT regimen (SAPT for 5 months + OAC) vs. Standard DAPT regimen (DAPT for 2 months + SAPT until 11 months + OAC)	Edoxaban 60 mg qd monotherapy vs. Edoxaban 60 mg qd plus clopidogrel	Edoxaban 60 mg qd monotherapy vs. Edoxaban + SAPT (aspirin or P2Y12I)
**FOLLOW-UP DURATION**	30 months	24 months (mean follow-up)	12 months	21 months	12 months
**PRIMARY ENDPOINT**	Composite of all-cause death, MI, stroke, or systemic embolism	- Primary efficacy endpoint: composite of stroke, systemic embolism, MI, unstable angina requiring revascularization, or death from any cause - Primary safety endpoint: ISTH major bleeding	- First co-primary endpoint: NACE (death, MI, stroke, and BARC 3 or 5 bleeding) - Second co-primary endpoint: MACCE (death, MI, or stroke) - Third co-primary endpoint: major or clinically relevant nonmajor bleedings (BARC type 2, 3, or 5)	ISTH major and clinically significant bleeding events	NACE, defined as composite of death from any causes, MI, stroke, systemic embolism, unplanned urgent revascularization, or major or clinically relevant nonmajor bleeding event by ISTH criteria
**PRIMARY ENDPOINT RATE**	**15.7% vs. 13.6%** (HR: 1.16, 95% CI: 0.79–1.72, *p* = 0.20)	- Primary efficacy endpoint: **4.14% vs. 5.75%** (HR: 0.72, 95% CI: 0.55–0.95, *p* < 0.001) - Primary safety endpoint: **1.62% vs. 2.76%** (HR: 0.59, 95% CI: 0.39–0.89, *p* = 0.01)	- First co-primary endpoint: **8.0% vs. 9.6%** (HR: 0.83, 95% CI: 0.60–1.15, *p* = 0.26) - Second co-primary endpoint: **5.9% vs. 6.7%** (HR: 0.88, 95% CI: 0.60–1.30, *p* = 0.53) - Third co-primary endpoint: **9.9% vs. 11.7%** (HR: 0.83, 95% CI: 0.62–1.12, *p* = 0.25)	**1.67% vs. 4.28%** (HR: 0.39, 95% CI: 0.08–2.02, *p* = 0.26)	**6.8% vs. 16.2%** (HR: 0.44, 95% CI: 0.30–0.65, *p* < 0.001)
**ANY BLEEDING RATE**	* 7.8% vs. 10.4% (HR: 0.73, 95% CI: 0.44–1.20, *p* = 0.22)	**7.22% vs. 12.72%** (HR: 0.58, 95% CI: 0.47–0.71)	^†^ **9.9% vs. 11.7%** (HR: 0.83, 95% CI: 0.62–1.12, *p* = 0.25)	^§^ **1.67% vs. 4.28%** (HR: 0.39, 95% CI: 0.08–2.02, *p* = 0.26)	**9.9% vs. 20.1%** (HR: 10.2, 95% CI: 5.73–14.67)
**ANY STENT THROMBOSIS RATE**	**0.58% vs. 0.0%**	**0.0% vs. 0.0%**	**0.4% vs. 0.5%** (HR: 0.72, 95% CI: 0.16–3.21, *p* = 0.66)	**0.0% vs. 0.0%**	**0.0% vs. 0.0%**
**ALL-CAUSE MORTALITY RATE**	**11.6% vs. 9.0%** (HR: 1.30, 95% CI: 0.82–2.10, *p* = 0.27)	**1.85% vs. 3.37%** (HR: 0.55, 95% CI: 0.38–0.81)	**3.7% vs. 4.1%** (HR: 0.90, 95% CI: 0.55–1.47, *p* = 0.67)	**^‡^ 1.66% vs. 0.86%** (HR: 1.99, 95% CI: 0.18–21.9, *p* = 0.58)	**0.6% vs. 0.7%** (HR: 1.29, 95% CI: 0.29–5.76)

* In the OAC-ALONE trial, any bleeding rate is reported as ISTH major bleedings. ^†^ In the MASTER-DAPT trial, any bleeding rate is reported as BARC 2, 3, or 5 bleedings. ^§^ In the PRAEDO-AF trial, any bleeding rate is reported as ISTH major or clinically significant bleedings. **^‡^** In the PRAEDO-AF trial, the mortality is reported only for non-cardiovascular death. Abbreviations: ACS, acute coronary syndrome; AF, atrial fibrillation; BARC, Bleeding Academic Research Consortium; CABG, coronary artery bypass graft; CAD, coronary artery disease; CAG, coronary angiography; CCTA, coronary computed tomography; DAPT, dual antiplatelet therapy; DES = drug-eluting stent; HBR = high bleeding risk; HBR = high bleeding risk; HR = hazard ratio; ISTH = International Society on Thrombosis and Haemostasis; MACCE, major adverse cardiac and cerebrovascular event; MI, myocardial infarction; NACE, net adverse clinical event; OAC, oral anticoagulation therapy; PCI, percutaneous coronary intervention; SAPT, single antiplatelet therapy; VKA, vitamin K antagonist.

## Data Availability

All data underlying this article will be shared on reasonable request to the corresponding author.

## Abbreviations

The following abbreviations are used in this manuscript:

PCI	Percutaneous Coronary Intervention
CAD	Coronary Artery Disease
DAPT	Dual Antiplatelet Therapy
AF	Atrial Fibrillation
OAC	Oral Anticoagulant
TAT	Triple Antithrombotic Therapy
DAT	Dual Antithrombotic Therapy
DES	Drug-Eluting Stent
ST	Stent Thrombosis
TLF	Target Lesion Failure
CABG	Coronary Artery Bypass Graft
ESC	European Society of Cardiology
STEMI	ST-Elevation Myocardial Infarction
NSTE-ACS	Non-ST-Elevation Acute Coronary Syndrome
UFH	Unfractionated Heparin
TRA	Transradial Access
TFA	Transfemoral Access
ACS	Acute Coronary Syndrome
BMS	Bare Metal Stent
HBR	High Bleeding Risk
CCS	Chronic Coronary Syndrome
MI	Myocardial Infarction
ZES	Zotarolimus-Eluting Stent
MACE	Major Adverse Cardiovascular Event
TVR	Target Vessel Revascularization
TLR	Target Lesion Revascularization
NACE	Net Adverse Cardiovascular Event
CRNMB	Clinically Relevant Nonmajor Bleeding
IVUS	Intravascular Ultrasound
OCT	Optical Coherence Tomography
MSA	Minimum Stent Area
TVF	Target Vessel Failure
MACCE	Major Adverse Cardiovascular and Cerebral Event
ACC	American College of Cardiology
AHA	American Heart Association
CKD	Chronic Kidney Disease
PPI	Proton Pump Inhibitor
DOAC	Direct Oral Anticoagulant
VKA	Vitamin K Antagonist
APT	Antiplatelet Therapy
SAPT	Single Antiplatelet Therapy
